# Role of functional mapping on Gallium-68 perfusion positron emission tomography and computed tomographic imaging (PET/CT) to assess the risk of long-term radiation-induced lung toxicity after stereotactic body radiation therapy

**DOI:** 10.1016/j.phro.2025.100786

**Published:** 2025-05-17

**Authors:** François Lucia, David Bourhis, Frédérique Blanc-Béguin, Gaëlle Goasduff, Mohamed Hamya, Simon Hennebicq, Maëlle Mauguen, Romain Floch, Margaux Geier, Ulrike Schick, Maëlys Consigny, Olivier Pradier, Grégoire Le Gal, Pierre-Yves Salaun, Vincent Bourbonne, Pierre-Yves Le Roux

**Affiliations:** a Radiation Oncology Department, University Hospital, Brest, France; b LaTIM, INSERM, UMR 1101, University of Brest 29200 Brest, France; c Service de médecine nucléaire, CHRU de Brest, Brest, France; d Medical Oncology Department, University Hospital, Brest, France; e GETBO, INSERM, UMR1304, Université de Bretagne Occidentale, Brest, France; f Direction de la Recherche Clinique et de l’Innovation (DRCI), CHU Brest, Brest, France; g Department of Medicine, Ottawa Hospital Research Institute at the University of Ottawa, Ottawa, Canada; h Centre d’investigation Clinique CIC 1412, Centre Hospitalier Régional et Universitaire de Brest, France

**Keywords:** Lung cancer, Stereotactic body radiation therapy, Gallium-68 perfusion PET/CT, Radiation-induced lung toxicity

## Abstract

**Background and purpose:**

To compare the performance of anatomic and functional dosimetric parameters based on Gallium-68 lung perfusion positron emission tomography and computed tomographic imaging (PET/CT) imaging to predict the risk of symptomatic long-term radiation-induced lung toxicity (RILT) in patients with lung tumors treated with stereotactic body radiation therapy (SBRT).

**Materials and methods:**

We have performed a prospective study in patients treated with SBRT. Mean dose (MD) and volumes receiving xGy were calculated in five lung volumes: the conventional anatomical volume (AV) delineated on CT images, three lung functional volumes defined on lung perfusion PET imaging (FV50%, FV70%, FV90%, i.e. the minimal volume containing 50 %, 70 % and 90 % of the total activity within the AV), and a low functional volume (LFV = AV-FV90%). The primary endpoint of this analysis was grade ≥2 long-term RILT at 12 months as assessed with NCI CTCAE v.5. The predictive value of anatomical and functional dose volume parameters was evaluated by comparing patients with and without long-term RILT.

**Results:**

Out of the 59 patients included, 50 were still alive at 12 months and 9 (18 %) had grade ≥2 long-term RILT. The MD and the VxGy in the AV and LFV were not statistically different in patients with and without long-term RILT (p > 0.05). All functional parameters in FV50% and FV70% were significantly higher in long-term RILT patients (p < 0.05).

**Discussion:**

The predictive value of PET perfusion-based functional parameters outperforms the standard CT-based dose-volume parameters for the risk of grade ≥2 long-term RILT.

## Highlights

• SBRT is a real alternative treatment for inoperable lung tumors but remains associated with significant pulmonary toxicity.
• Perfusion PET/CT imaging is a very attractive test for functional lung avoidance during radiotherapy planning.
• This prospective study compare anatomical and functional dosimetric parameters to predict the risk of long-term RILT.
• The predictive value of functional parameters outperforms the standard parameters for the risk of grade ≥2 long-term RILT.
• Functional parameters could be useful to guide radiotherapy planning to reduce the risk of long-term RILT.

## 1 Introduction

Over recent years, stereotactic body radiotherapy (SBRT) has demonstrated efficacy in treating early-stage non-small cell lung cancer and lung metastases with less toxicity than conventionally fractionated RT [1,2]. Nevertheless, SBRT is still associated with a late radiation-induced lung toxicity (RILT) requiring medical intervention (grade ≥2 according to the National Cancer Institute Common Terminology Criteria for Adverse Events [CTCAE] version 5.0 grading scale) of the order of 15–20 % [3].

The prediction models in the literature used standard dosimetric parameters based on the anatomic lung. These models had moderate performance with Area under curve (AUC) values of 0.63 to 0.77 [4,5] indicating that the predictive accuracy needs to be improved. Development of an accurate prediction model for RILT is an important challenge [3].

Current treatment planning dosimetric constraints are applied on the anatomical lung volume defined on CT images, and simplistically assume that lung tissue is functionally homogeneous. However, multiple studies have now demonstrated that patients’ lung function is spatially heterogeneous, especially in patients with lung tumors due to tumor or tobacco-related comorbidities [6,7]. Thus, despite respect of standard lung dose constraints, the RILT rate remains significant [3].

One strategy for mitigating RILT could be to preferentially spare the radiation dose to highly functional regions of the lung. Various imaging techniques have been proposed for lung functional mapping and radiotherapy planning. The majority of studies used perfusion single-photon emission computed tomography (SPECT) imaging and demonstrated the ability of functional lung optimized planning techniques to spare regions of functional lung. However, the predictive value of functional dose-volume parameters based on SPECT images on RILT has not been clearly demonstrated [8].

Lung perfusion positron emission tomography and computed tomographic imaging (PET/CT) is an emerging modality for regional lung function assessment [[9], [10], [11]]. As compared with conventional lung scintigraphy, ^99m^Tc is substituted with ^68^Ga to label Macro aggregated albumin (MAA) particles. PET/CT is a vastly superior technology for image acquisition, with an approximately 100 times higher sensitivity, higher spatial resolution (3–4 mm with PET compared with 15 mm with SPECT), and greater access to respiratory-gated acquisition [12,13].

In a previous analysis, we found that the predictive value of PET perfusion-based functional parameters outperforms the standard CT-based dose volume parameters for the risk of grade ≥2 acute RILT [14]. However, RILT is generally divided into an acute phase (acute RILT), within 3–6 months after RT, often referred to as radiation-induced pneumonitis, and a late phase (long-term RILT), often referred to as radiation-induced pulmonary fibrosis, which is a chronic toxicity with a long-term impact on the patient's quality of life [15].

The aim of this analysis was to compare the performance of anatomic and functional dosimetric parameters based on lung perfusion PET/CT imaging to predict the risk of symptomatic long-term RILT in patients with lung tumors treated with SBRT.

## 2 Material and methods

### 2.1 Study design and participants

The PEGASUS trial is a single-center prospective study [16]. The eligible study population consisted of patients aged >18 years planned to be treated in the radiotherapy department of our institute, with SBRT for primary or secondary lung tumors. Exclusion criteria included the inability to give informed consent, patients under guardianship or curatorship, pregnant or breastfeeding women, and contra-indication to the administration of human albumin macroaggregates. The study was approved by the Nord Ouest IV Ethics Committee (ID RCB: 2021-002224-20) and registered in ClinicalTrial.gov registry (NCT04942275). Written informed consent was obtained from all participants.

### 2.2 Radiation therapy

A respiratory-sorted 4-dimensional computed tomography (4DCT) data set was generated using the planning CT (Siemens, Somatom) coupled with Varian real-time position management (RPM) gating system (Varian Medical Systems, Palo Alto, CA, United States). From the respiratory-sorted image phases, average (AVG) and maximum intensity projection (MIP) series were reconstructed. The internal target volume was delineated on the basis of a four-dimensional planning CT scan to take account for tumour motion on a MIM Maestro v7.2.3 (MIM Software, Cleveland, Ohio, USA) workstation. The internal target volume (ITV) was expanded 3 mm in all plans to create the planning target volume (PTV). Treatment plan was performed on the Pinnacle V16.2 planning system (Philips Medical Systems (Cleveland), Inc.) using the AVG series. Treatment was planned and delivered with modulated arc therapy (VMAT) using a TrueBeamTM STX 2.0 linac linear accelerator (Varian Medical Systems, Palo Alto, CA, United States) equipped with Varian RPM gating system with a prescription dose of 48 to 60 Gy in 3 to 8 fractions depending on proximity to organs at risk (OAR) [14] (Supplemental data A). As per the protocol of this pilot study, and given that doses to the target volume and the OARs were respected, either the anatomical plan or functional plan could be delivered to the patient.

### 2.3 Lung perfusion PET/CT

All patients underwent lung perfusion PET/CT scan acquired on a digital Biograph Vision 600 PET/CT scanner (Siemens Healthineers, Knoxville, TN, United States) [16] (Supplemental data A).

The lung volumes were delineated using MIM Maestro v7.2.3 (MIM Software, Cleveland, Ohio, USA). An automatic contouring of the whole lung anatomical volume (AV) was initially performed based on Hounsfield unit value and then visually adjusted to match normal contours if required. Within the AV, three lung functional volumes were defined using an automated relative to whole lung function segmentation method, delineating the minimal volume containing 50 % (FV50%), 70 % (FV70%) and 90 % (FV90%) of the total activity within the AV, respectively [17]. We also defined a low functional lung volume (LFV) as follows: LFV = AV-FV90% (approximately 25 % of the AV containing 10 % of lung function [17].

### 2.4 PET/CT and planning CT registration

Functional volumes were defined on PET, and AV was defined the CT component of PET (CT_PET_), then all volumes were stored associated with CT_PET_ in DICOM RT-STRUCT format. Dose distribution map was associated with the average of planning 4DCT (AVG) in DICOM RT-DOSE format. Second, AVG and CT_PET_ were registered, with a rigid followed by a deformable registration using MIM Maestro v7.2.3 (MIM Software, Cleveland, Ohio, USA) and the VoxAlign Deformation Engine, a constrained, intensity based free-form deformable registration algorithm [18,19], in order to take into account differences in terms of respiratory cycle. Finally, lung functional volumes and AV were transferred to AVG, allowing optimization to lung volumes and dose-volume analysis.

### 2.5 End-point: RILT evaluation

RILT was assessed prospectively by a radiation oncologist, without access to dose statistics for the functional pulmonary volume, every 3 months until 12 months after completion of SBRT, based on clinical and imaging evaluation according to NCI CTCAE version 5.0 (pneumonitis or fibrosis) [3,20].

The main endpoint of the present analysis was grade ≥2 long-term RILT at 12 months as assessed with NCI CTCAE version 5.0 [3,20].

### 2.6 Statistical analysis

Clinical parameters included in the analysis were age, sex, Eastern Cooperative Oncology Group (ECOG) performance status (PS), smoking history including pack-years, histology (primary vs secondary), tumor volume, spirometry including forced expiratory volume in one second (FEV1) and diffusion capacity of the lung for carbon monoxide (DLCO).

Dosimetric parameters included dose fractionation, dose received by 99 % volume of PTV (D99%), PTV maximum dose (DPTVmax), PTV minimum dose (DPTVmin) and PTV mean dose (DPTVmean), mean dose (MD) to lung volume, percentage volume of lung getting 5 to 30 Gy dose (Vx) in 5-Gy increments.

MD and percentage of volume getting 5 to 30 Gy dose (VxFVy) were extracted from five lung volumes: the AV, three lung functional volumes (FV50%, FV70%, FV90) and the LFV. We also reported the Biologically Effective Dose (BED), using the formula BED = D x (1 + [d/(α/β)]), where variables are as follows: d = Dose per fraction, in Gy, D = Total dose (number of fractions x dose per fraction), in Gy, and α/β ratio = 3 Gy for the lungs.

Population characteristics were compared between RILT and non-RILT groups by Wilcoxon tests for quantitative variables and by Fisher's Exact tests for qualitative variables. The Wilcoxon test was also used to compare dose-volume parameters between RILT and non-RILT groups. The area under the curve (AUC) of each ROC curve for the five lung volumes as predictors of RILT (AV, FV50%, FV70%, FV90%, LFV) was calculated.

We also performed a time dependent analysis, using Kaplan-Meier curves, of the probability of grade ≥2 RILT occurrence between groups classified as high risk and low risk defined according to cutoff values obtained with Younden indices of previous ROC curves. In this analysis, patients lost to follow-up or who died are censored. Analyses were performed using SAS 9.4. Multivariate statistical tests were not performed due to the small sample size and low number of patients experiencing toxicity. However, we performed statistical significance adjustment, due to multiple testing, using the Benjamin-Hochberg false discovery rate (FDR) procedure with a threshold adjusted p-value of 5 %.

## 3 Results

### 3.1 Study population

Between July 2021 and January 2022, 60 consecutive patients who consented to participate in the study and met inclusion criteria with primary or secondary lung tumors were enrolled into this prospective study. Lung SBRT was not performed in one patient with NSCLC due to a brain progression of the disease, for which systemic treatment was initiated. Out of the 59 patients, 50 patients were alive at 12 months. Patient characteristics and dose target volumes are presented in Table 1.

**Table 1:** Patient characteristics.

		**Total(N = 50)**	**Grade 0**–**1(N = 41)**	**Grade ≥ 2(N = 9)**	**P value**	**Adjusted P value**
Age	No. (No. missing)	50 (0)	41 (0)	9 (0)	0.55	0.65
	Median (q1;q3)	69.5 (63.0;72.0)	68.0 (63.0;72.0)	71.0 (63.0;75.0)		
Gender	Male	25 (50 %)	18 (44 %)	7 (78 %)	0.14	0.24
	Female	25 (50 %)	23 (56 %)	2 (22 %)		
Tobacco	*Missing*	4 (8 %)	3 (7 %)	1 (11 %)	0.16	0.26
	Active smoker	13 (28 %)	13 (34 %)	0 (0.0 %)		
	Former smoker	21 (46 %)	16 (42 %)	5 (62 %)		
	Non smoker	12 (26 %)	9 (24 %)	3 (38 %)		
Pack-years smoking	No. (No. missing)	28 (22)	26 (15)	2 (7)	0.43	0.55
	Median (q1;q3)	40.0 (20.0;57.5)	40.0 (20.0;60.0)	30.0 (20.0;40.0)		
Asbestos exposure	*Missing*	6 (12 %)	6 (15 %)	0 (0 %)	0.57	0.65
	No	39 (89 %)	30 (86 %)	9 (100 %)		
	Yes	5 (11 %)	5 (14 %)	0 (0 %)		
Weight	N (N missing)	45 (5)	37 (4)	8 (1)	0.24	0.35
	Median (q1;q3)	60.0 (56.0;79.0)	59.0 (54.0;76.0)	73.5 (58.0;93.5)		
Performance status	N (N missing)	48 (2)	39 (2)	9 (0)	0.98	1.00
	Median (q1;q3)	1.0 (0.0;1.0)	1.0 (0.0;1.0)	0.0 (0.0;1.0)		
COPD	No	35 (70 %)	28 (68 %)	7 (78 %)	0.71	0.77
	Yes	15 (30 %)	13 (32 %)	2 (22 %)		
Asthma	No	47 (94 %)	39 (95 %)	8 (89 %)	0.46	0.57
	Yes	3 (6 %)	2 (5 %)	1 (11 %)		
Previous pulmonary embolism	No	46 (92 %)	37 (90 %)	9 (100 %)	1.00	1.00
	Yes	4 (8 %)	4 (10 %)	0 (0 %)		
Other(s) comorbiditie(s)	No	6 (12 %)	5 (12 %)	1 (11 %)	1.00	1.00
	Yes	44 (88 %)	36 (88 %)	8 (89 %)		
Thoracic surgery	No	36 (72 %)	28 (68 %)	8 (89 %)	0.41	0.54
	Yes	14 (28 %)	13 (32 %)	1 (11 %)		
Localization	Central	6 (12 %)	5 (12 %)	1 (11 %)	1.00	1.00
	Peripheral	44 (88 %)	36 (88 %)	8 (89 %)		
ITV volume in cc	Median (q1;q3)	5.9 [3.0; 9.9]	5.9 [2.8; 9.9]	5.9 [3.5; 9.0]	0.71	0.77
PTV volume in cc	Median (q1;q3)	12.9 [8.2; 21.3]	13.1 [8.1; 21.6]	12.6 [9.9; 20.5]	0.76	0.80
SBRT dose	54 Gy	18 (36 %)	16 (39 %)	2 (22 %)	0.48	0.59
	48 Gy 60 Gy	26 (52 %) 6 (12 %)	20 (49 %) 5 (12 %)	6 (67 %) 1 (11 %)		

Abbreviations: COPD = chronic obstructive pulmonary disease; SBRT = stereotactic body radiation therapy.

### 3.2 Toxicity

Out of the 50 patients, 9 (18 %) had grade ≥ 2 long-term RILT according to the CTCAE version 5.0 criteria. 45 (76 %) patients had some nonspecific radiological changes on CT scan. Two (4 %) patient developed grade 3 long-term RILT. There were no deaths attributed to RILT. No patient had a rib fracture or thoracic pain. Other symptom reported by the patients were asthenia in 3 (6 %) patients, all grade 1.

### 3.3 Comparison of dosimetric parameters in patients with and without long-term RILT

Dosimetric parameters in patients with and without long-term RILT are presented in Tables 2 and S1.

**Table 2:** Dosimetric parameters.

	**Total (N = 50)** **Median IQR**	**Grade 0**–**1 (N = 41)** **Median IQR**	**Grade ≥ 2 (N = 9)** **Median IQR**	**P value**	**Adjusted p value**
MD AV (Gy)	2.9 (2.0;4.3)	2.8 (1.9;3.8)	4.3 (2.5;4.6)	0.10	0.20
MD LFV (Gy)	2.3 (1.0;4.3)	2.6 (0.9;4.4)	2.1 (1.2;2.5)	0.65	0.73
MD FV50% (Gy)	3.1 (1.6;4.2)	2.7 (1.4;3.7)	4.8 (4.1;5.5)	0.001	0.006
MD FV70% (Gy)	3.1 (1.9;4.1)	2.7 (1.8;3.8)	4.8 (3.7;5.6)	0.004	0.013
MD FV90% (Gy)	2.8 (2.1;4.5)	2.8 (1.8;3.6)	4.8 (3.1;5.3)	0.014	0.031
MD AV (Gy) BED	7.6 (6.0;11.3)	7.5 (5.4;10.2)	9.5 (6.8;11.4)	0.16	0.26
MD LFV (Gy) BED	5.9 (1.8;11.4)	6.2 (1.8;11.7)	4.1 (2.8;6.2)	0.48	0.59
MD FV50% (Gy) BED	6.2 (3.4;11.3)	5.6 (3.0;9.3)	14.3 (11.6;16.7)	<0.001	0.005
MD FV70% (Gy) BED	7.4 (4.9;10.2)	6.5 (4.1;8.3)	12.2 (10.5;16.6)	<0.001	0.005
MD FV90% (Gy) BED	8.1 (5.3;9.8)	7.1 (4.3;9.0)	12.3 (8.2;15.1)	0.006	0.017
V5Gy AV (%)	13.6 (9.7;20.4)	12.4 (9.1;18.4)	15.8 (12.6;23.3)	0.14	0.24
V5Gy LFV (%)	9.6 (3.7;22.1)	10.1 (3.7;22.1)	8.3 (5.9;14.7)	0.73	0.77
V5Gy FV50% (%)	16.1 (8.7;21.3)	12.5 (6.6;18.7)	23.2 (17.8;25.3)	0.008	0.022
V5Gy FV70% (%)	14.4 (10.2;20.0)	12.8 (9.4;17.9)	24.0 (15.5;30.1)	0.009	0.022
V5Gy FV90% (%)	14.0 (10.2;18.6)	13.3 (10.0;17.3)	24.1 (13.4;27.4)	0.031	0.066
V5Gy AV (%) BED	15.9 (12.5;22.5)	15.6 (11.6;21.3)	16.3 (15.5;28.0)	0.21	0.33
V5Gy LFV (%) BED	13.7 (4.2;24.2)	13.7 (4.2;24.3)	13.7 (9.1;15.5)	0.73	0.77
V5Gy FV50% (%) BED	13.1 (7.6;23.4)	10.2 (7.4;19.9)	26.0 (21.6;29.3)	0.002	0.009
V5Gy FV70% (%) BED	13.8 (9.1;22.9)	11.6 (8.9;19.4)	25.6 (19.5;28.6)	0.002	0.009
V5Gy FV90% (%) BED	15.5 (9.5;21.1)	13.9 (9.3;19.5)	23.9 (17.5;30.6)	0.008	0.022
V20Gy AV (%)	3.0 (2.0;5.1)	2.7 (2.0;4.7)	5.1 (2.2;6.6)	0.18	0.28
V20Gy LFV (%)	2.6 (0.4;5.8)	2.8 (0.4;5.8)	0.6 (0.3;3.4)	0.38	0.50
V20 Gy FV50% (%)	3.5 (1.2;4.9)	2.2 (1.0;3.9)	6.4 (4.7;8.9)	<0.001	0.005
V20 Gy FV70% (%)	3.4 (1.4;5.6)	2.8 (1.3;4.2)	6.8 (4.0;8.2)	0.004	0.013
V20 Gy FV90% (%)	3.4 (1.7;5.1)	3.2 (1.5;4.6)	6.9 (2.9;7.5)	0.026	0.057
V20Gy AV (%) BED	6.8 (4.7;10.7)	6.7 (4.6;9.2)	9.7 (7.1;14.2)	0.11	0.21
V20Gy LFV (%) BED	6.0 (1.4;11.1)	6.4 (1.4;11.3)	3.3 (1.4;7.1)	0.38	0.50
V20 Gy FV50% (%) BED	5.5 (2.5;9.9)	5.1 (2.3;6.6)	12.4 (9.7;16.7)	<0.001	0.005
V20 Gy FV70% (%) BED	6.0 (3.5;9.2)	5.4 (3.4;7.7)	11.9 (9.0;13.2)	<0.001	0.005
V20 Gy FV90% (%) BED	6.8 (4.0;9.7)	5.6 (3.5;7.7)	10.2 (9.2;13.8)	0.004	0.013

Abbreviations: AV = anatomic volume; BED = biologically effective dose; EQD2 = equivalent dose in 2-Gy fractions; FV = functional volume; LFV = low functional volume; MD = mean dose; VxGy = percentage of lung volumes receiving xGy.

After FDR adjustment, median (IQR) MD in the AV and LFV volumes were not statistically different in patients with and without long-term RILT with p = 0.20 and p = 0.73, respectively. In contrast, median MD was significantly higher in the FV50%, FV70% and FV90% volumes in patients with long-term RILT with p = 0.006, p = 0.013 and p = 0.031, respectively.

After FDR adjustment median V20Gy in the AV and LFV volumes were not statistically different in patients with and without long-term RILT with p = 0.28 and p = 0.50, respectively. In contrast, median V20Gy was significantly higher in the FV50% and FV70% volumes, but not in the FV90% volume, in patients with long-term RILT, with p = 0.005, p = 0.013 and p = 0.057, respectively.

The results in BED were slightly higher (See Tables 2 and S1).

Among the clinical characteristics tested, none was found to be significantly higher in patients with long-term RILT grade ≥2 (Table 1).

**Fig. 1:**
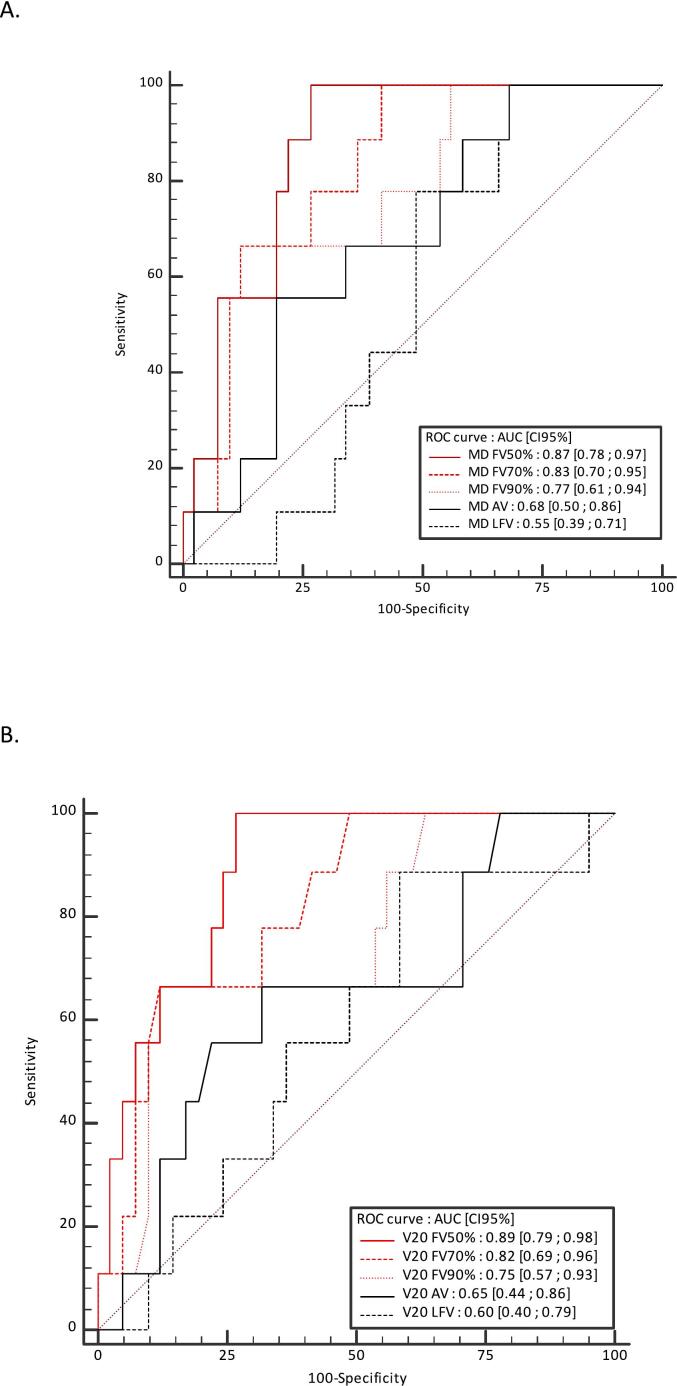
Receiver operating characteristic (ROC) curves to assess the ability of the mean dose (A) and V20Gy (B) of anatomic volume (AV), low functional volume (LFV), and the minimal volume containing 50% (FV50%), 70% (FV70%), and 90% (FV90%) of the total activity within the AV to discriminate between patients with and without long-term symptomatic radiation-induced lung toxicity. *Abbreviations:* AUC = area under the curve; MD = mean dose.

**Fig. 2:**
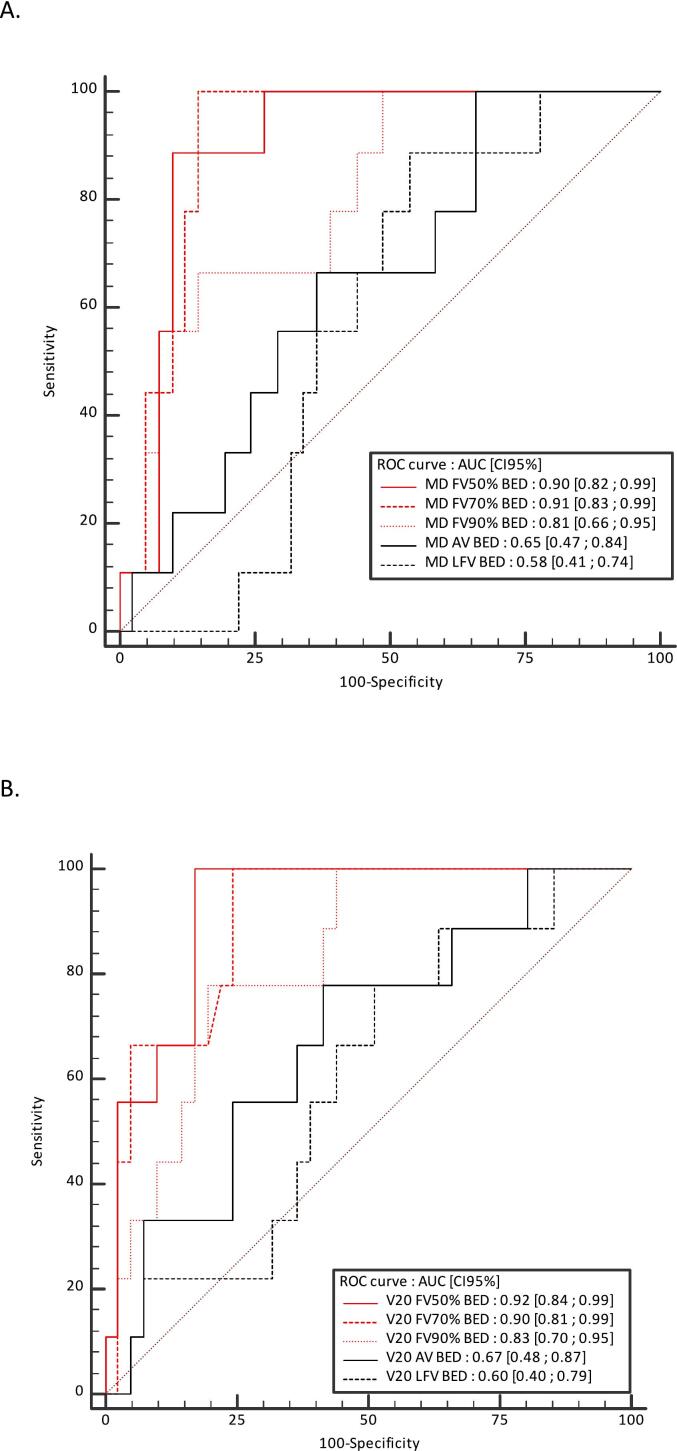
Receiver operating characteristic (ROC) curves to assess the ability of the mean dose (A) and V20Gy (B) in biologically effective dose (BED) of anatomic volume (AV), low functional volume (LFV), and the minimal volume containing 50% (FV50%), 70% (FV70%), and 90% (FV90%) of the total activity within the AV to discriminate between patients with and without long-term symptomatic radiation-induced lung toxicity. *Abbreviations:* AUC = area under the curve; MLD = mean dose.

ROC curves assessing the ability of the MD AV, LFV, FV50%, FV70% and FV90% and V20 AV, LFV, FV50%, FV70% and FV90% to discriminate between patients with and without long-term symptomatic RILT are shown in Fig. 1, Fig. 2. AUCs (CI 95 %) for MD AV, LFV, FV50%, FV70% and FV90% were 0.68 (0.50–0.86), 0.55 (0.39–0.71), 0.87 (0.78–0.97), 0.83 (0.70–0.95), 0.77 (0.61–0.94), respectively. AUCs for V20Gy AV, LFV, FV50%, FV70% and FV90% were 0.65 (0.44–0.86), 0.60 (0.40–0.79), 0.89 (0.79–0.98), 0.82 (0.69–0.96), 0.75 (0.57–0.93), respectively. The results in BED were slightly higher (See Fig. 2).

### 3.4 Time-dependent analysis of the occurrence of grade ≥2 RILT

**Fig. 3:**
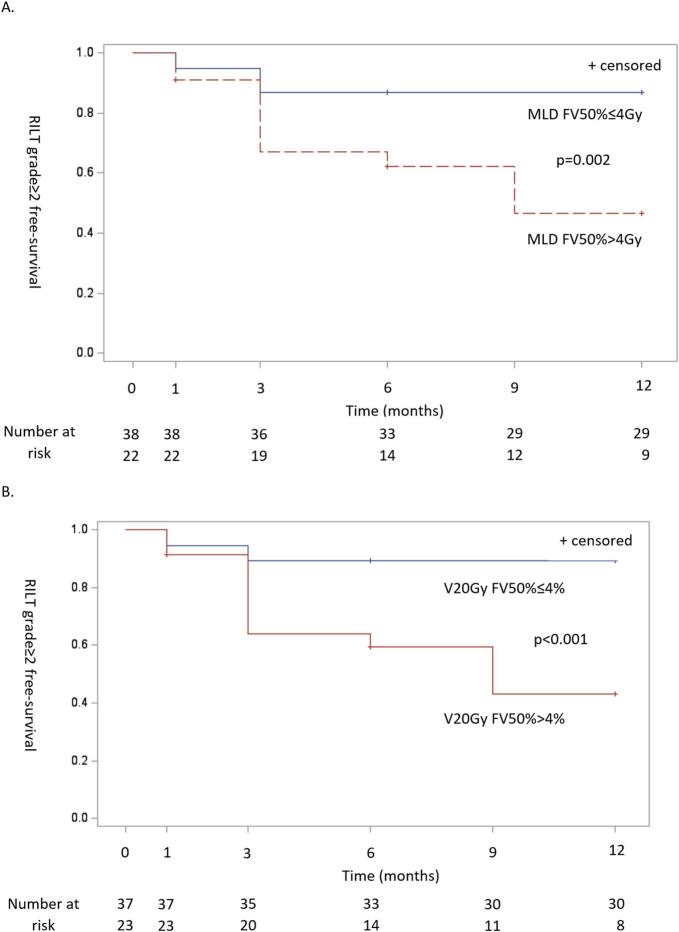
Risk of ≥grade 2 radiation-induced lung toxicity according to (A) MD FV50% (cut-off of 4 Gy) and (B) V20Gy FV50% (cut-off of 4 %) over time.

Based on ROC analysis, MD FV50% and V20 FV50% were the most predictive parameters of long term RILT. The most discriminant values of functional dosimetric parameters to separate patients with low- or high-risk grade ≥2 RILT occurrence were 4 Gy for MD FV50% and 4 % for V20Gy FV50%, respectively. Time-dependant analysis of the occurrence of grade ≥2 RILT were significantly different for MD and V20Gy FV50% (p = 0.002 and p < 0.001, respectively) and are presented in Fig. 3.

## 4 Discussion

In this prospective study, functional dose parameters based on lung perfusion PET/CT imaging outperformed anatomical lung parameters to predict the risk of long-term RILT grade ≥2 after SBRT.

The incidence of symptomatic grade ≥2 long term RILT in our study (18 %) was in line with that of the published literature, which is around 15–20 % [3,21,22]. Because of the difficulty in differentiating radiation pneumonitis and fibrosis on the basis of clinical and imaging findings [23,24], they are referred to as acute or long term « radiation-induced lung toxicity.

It is important to note that the presence or absence of acute RILT at 3 months does not allow to prejudge the presence of long term RILT. In our study, of the 10 patients with grade ≥2 long-term RILT, 3 had no acute toxicity at 3 months. In contrast, 3 patients with acute RILT and evaluable at 12 months had no long term toxicity, with regression of their symptoms after treatment with corticosteroids. Our result underlines the difference between acute and long-term RILT, and the need to improve prediction of long-term toxicity with the risk of sequelae for the patient. Although corticosteroids are the cornerstone of the clinical management of radiation pneumonitis, advanced knowledge of the molecular mechanisms underlying lung injury has led to the development of other promising pharmaceuticals to treat radiation fibrosis [25,26]. As long-term use of corticosteroids can have adverse side effects and are inefficient, the prediction of long-term RILT using a non-invasive tool is clinically significant to allow prevention or earlier and more appropriate therapeutic management.

Functional lung avoidance radiotherapy planning is an emerging concept to reduce the RILT [6,8]. In our study, all dose volume parameters were significantly higher in the FV50% and FV70% volumes in patients with long-term RILT (p < 0.05). In contrast, no significant difference was observed with the AV and the LFV. Furthermore, AUCs of MD and V20Gy in all FV were between 0.75 and 0.89 vs 0.65 to 0.68 in the AV and 0.55 to 0.58 in LFV. No significant differences were observed between the anatomical (7/43 with RILT grade ≥2) and functional (2/7 with RILT grade ≥2) plans groups. This finding could be attributed to a limited sample size and optimization that was not guided by established dose constraints on functional lung volumes.

This analysis confirmed our previous results at 3 months [14]. FV70% and FV50% are the two functional volumes that are most predictive of grade 2 or higher acute and late RILT. These two functional volumes seem to have a better predictive power for late toxicity compared to acute RILT, with AUCs of approximately 0.9 for late toxicity versus 0.8 for acute toxicity. The optimal cut-off values to distinguish patients at high risk of acute or late RILT are, for the mean dose to FV50% and FV70%, 3 Gy in physical dose and 11 Gy and 9 Gy in BED, respectively. As outlined in a recent systematic review and meta-analysis on functional lung avoidance radiotherapy [8], no study had previously demonstrated a statistically significant better predictive value of functional versus anatomical lung volumes for RILT risk [8]. We adopted a different methodology on several points. First, we assessed patients treated with SBRT while all previous studies were performed in patients treated with conventional RT. Second, we used PET rather than SPECT imaging for lung functional volume mapping. PET/CT is a vastly superior technology for image acquisition, with an approximately 100 times higher sensitivity, higher spatial resolution, and greater access to respiratory-gated acquisition [9,10], allowing a more precise functional lung volumes mapping and a better co-registration with radiation plans. Third, we used an automated relative to whole lung function threshold segmentation method for lung functional volumes delineation. This delineation method provides reliable and reproducible functional lung volumes, and is clinically meaningful to clinicians [17].

ROC curves analysis provides insight on the potential use of lung perfusion PET/CT images for lung avoidance SBRT planning. For instance, the rate of grade ≥2 RILT was 3.0 % in patients with MD in the FV50% <4 Gy, as compared with 47.1 % for patients with MD in the FV50% >4 Gy. The rate of grade ≥2 RILT was 3.1 % in patients with V20 FV50% <4%, as compared with 44.4 % in patients with V20 FV50% >4%. All functional parameters were able to separate patients at high versus low risk of developing grade ≥2 RILT over time, allowing a personalized optimization of radiation treatment planning according to the individual heterogeneity of regional lung function distribution. Moreover, the low functional lung volume was not predictive of long-term symptomatic RILT, opening up perspectives for further adaptation of radiation plans by irradiating these areas to better spare the functional lung volumes.

Our study has several limitations. Firstly, in this pilot study whose primary aim was to assess the feasibility of adapting the radiotherapy treatment plan to lung functional volumes, the number of patients was relatively small. The low power may explain why no statistical difference was observed in dose volumes parameters in the anatomical volumes. However, functional lung dose parameters based on lung perfusion PET/CT imaging clearly outperformed anatomical lung parameters to predict the risk of long-term RILT grade ≥2. Secondly, changes in pulmonary function testing (PFT) and impact on QoL were not available for the majority of patients. These changes would be interesting to analyze in a future trial in view of the results of previous studies [27,28]. Finally, there is a potential bias due to the challenge of differential diagnosis between RILT vs. progression of COPD or neoplasia.

Strengths of the study include the prospective design, with PET/CT scans systematically performed before initiation of SBRT. All patients had a close monitoring and follow up. RILT gradation was performed blindly, using the National Cancer Institute Common Terminology Criteria for Adverse Events, a widely accepted tool used for RILT evaluation.

In conclusion, functional dose parameters based on lung perfusion PET/CT outperformed standard CT parameters to predict the risk of long-term RILT grade ≥2 after lung SBRT. Functional lung avoidance planning may be a simple and non-invasive method to reduce radiation-induced lung sequelae, while delivering an optimal dose to the tumor and preserving others organs at risk. Randomized trials are now needed to determine whether a functional planning is superior to a conventional anatomical planning to reduced radiation-induced lung toxicity.

## 8 Consent to participate

The study was approved by the Nord Ouest IV Ethics Committee (ID RCB: 2021-002224-20) and registered in ClinicalTrial.gov registry (NCT04942275). Written informed consent was obtained from all participants.

## Author contribution

All authors contributed to the study conception and design. Material preparation, data collection and analysis were performed by François Lucia and Pierre-Yves Le Roux. The first draft of the manuscript was written by François Lucia and Pierre-Yves Le Roux and all authors commented on previous versions of the manuscript. All authors read and approved the final manuscript.

## Ethics approval

All procedures were performed in accordance with the principles of the 1964 Declaration of Helsinki and its later amendments or comparable ethical standards. The study design and exemption from informed consent were approved by the Institutional Review Board of Brest University Hospital.

## Funding

This study received funding from “la ligue contre le cancer” (CD29 and CD22). The sponsor had no role in the design, analysis, or interpretation of the results.

## Declaration of competing interest

The authors declare that they have no known competing financial interests or personal relationships that could have appeared to influence the work reported in this paper.

## Data Availability

The datasets generated during and/or analysed during the current study are available from the corresponding author on reasonable request.
