# Supplementary material for: Role of functional mapping on Gallium-68 perfusion positron emission tomography and computed tomographic imaging (PET/CT) to assess the risk of long-term radiation-induced lung toxicity after stereotactic body radiation therapy
Source: Phys Imaging Radiat Oncol. 2025 May 17;34:100786. doi: 10.1016/j.phro.2025.100786 (PMC12150183; doi:10.1016/j.phro.2025.100786)
Supplement: Supplementary Data 1 [file mmc1.docx]

*2.Material and methods*

*2.2 Radiation therapy*

A respiratory-sorted 4-dimensional computed tomography (4DCT) data set was generated using the planning CT (Siemens, Somatom) coupled with Varian real-time position management (RPM) gating system (Varian Medical Systems, Palo Alto, CA, United States). The 4D CT scans were acquired in helical mode and binned into 8 phases for image reconstruction. From the respiratory-sorted image phases, average (AVG) and maximum intensity projection (MIP) series were reconstructed. The internal target volume was delineated on the basis of a four-dimensional planning CT scan to take account for tumour motion on a MIM Maestro v7.2.3 (MIM Software, Cleveland, Ohio, USA) workstation. The internal target volume (ITV) was expanded 3 mm in all plans to create the planning target volume. The lung organ at risk (OAR) was defined as the volume of both lungs minus the volume of the ITV. Treatment plan was performed on the Pinnacle V16.2 planning system (Philips Medical Systems (Cleveland), Inc.) using the AVG series. Treatment was prescribed so that 99% of the PTV received at least 99% of the prescribed dose. The maximum dose at the PTV should be < 140% of the prescribed dose. All patients were planned to receive three fractions of 18 Gy each (total 54 Gy), or if the tumour was less than 2 cm from the chest wall, four fractions of 12 Gy each (total 48 Gy), or if the tumor was central or ultracentral, eight fractions of 7.5 Gy each (total 60 Gy). Treatment was planned and delivered with modulated arc therapy (VMAT). Density heterogeneity was taken into account by using the Collapsed Cone Convolution algorithm. Treatment was delivered under free breathing. Cone-beam computed tomography (CBCT) image was performed at the beginning of each session to position the patient, verifying that the identified/visible tumor volume on the image was included within the PTV contour. The CBCT, validated by the radiation oncologist, ensured that the target tumor was well encompassed within the PTV defined from the ITV. Expiration and inspiration thresholds of the gating window were defined using kV image. During treatment, kV image was performed every 15 seconds.

All patients had a treatment plan carried out in 2 stages. First, an anatomical plan was carried out, blinded to the PET results. Then, a functional plan, respecting the standard constraints applied during anatomical plan, but also incorporating “lung functional volume” constraints defined by pulmonary PET (FV50%, FV70% and FV90%), was carried out. For anatomical plan, the optimization was only performed to the anatomical lung. For functional plan, we have tried to reduce doses to the functional lung based on the results of anatomical plan. In the absence of previous studies, we had no particular dose constraints, so we tried to reduce doses to the functional lung as much as possible, while maintaining the doses obtained on the target volume and other OARs at the time of anatomical plan. As per the protocol of this pilot study, and given that doses to the target volume and the OARs were respected, either the anatomical plan or functional plan could be delivered to the patient. We only selected the functional plan if it allowed for equal or superior coverage of the target volumes, as well as equal or lower doses to organs at risk, and if the staging assessment (^18^F-FDG PET/CT and brain imaging) was less than 4 weeks old. However, in the majority of cases, due to the time required to optimize the functional plan to meet all dosimetric constraints, it was not ready within the allotted time of less than 4 weeks from the staging assessment. In fact, anatomical plan was used for 43 patients and the functional plan for the remaining 7.

*2.3 Lung perfusion PET/CT*

All patients underwent lung perfusion PET/CT scan acquired on a digital Biograph Vision 600 PET/CT scanner (Siemens Healthineers, Knoxville, TN, United States) ^16^. [68Ga]Ga-MacroAggregated Albumin (MAA) suspensions were prepared in the radiopharmacy unit using an automated process with a miniAIO® module, disposable cassettes from Trasis (Belgium) ^18^, and a commercial MAA kit used for 99mTc labelling. Patients were positioned supine on the PET/CT scanner with their arms raised. First, a low dose CT was acquired using 120 kV and 10mAs. Approximately 50 MBq of ^68^Ga-MAA were injected and PET images were acquired immediately after. Perfusion PET data was acquired covering the whole lungs in continuous bed motion mode at 1.9 mm/s, leading to an acquisition time of approximately 5 minutes. PET data was reconstructed using OSEM 3D algorithm with time of flight (ToF) and point spread function (PSF) correction (TrueX+TOF). PET images were corrected for random coincidence, scatter, deadtime, normalization, isotope decay and attenuation using CT data. The size of the transaxial reconstruction was 220 x 220 (voxel size = 3.3 x 3.3 x 3.3 mm^3^) with 4 iterations, 5 subsets and 4 mm Gaussian post-filtering.

The lung volumes were delineated using MIM Maestro v7.2.3 (MIM Software, Cleveland, Ohio, USA). An automatic contouring of the whole lung anatomical volume (AV) was initially performed based on Hounsfield unit value and then visually adjusted to match normal contours if required. Within the AV, three lung functional volumes were defined using an automated relative to whole lung function segmentation method, delineating the minimal volume containing 50% (FV50%), 70% (FV70%) and 90% (FV90%) of the total activity within the AV, respectively ^19^. We also defined a low functional lung volume (LFV) as follows: LFV=AV-FV90% (approximately 25% of the AV containing 10% of lung function ^19^.

*4.Discussion*

Besides improving the quality of images and the accuracy of lung functional volumes delineation, lung perfusion PET/CT imaging offers several advantages ^12,13^. Images are acquired immediately after administration of the radiotracer. There is therefore no waiting time in a dedicated waiting room. The acquisition duration is approximately 5 min. These make the test convenient for the patient and easy to implement in nuclear medicine departments. It is a simple and noninvasive test, with no contraindication or side-effects. The radiation dose is negligible as compared with that of radiation therapy.
