# Supplementary material for: Role of functional mapping on Gallium-68 perfusion positron emission tomography and computed tomographic imaging (PET/CT) to assess the risk of long-term radiation-induced lung toxicity after stereotactic body radiation therapy
Source: Phys Imaging Radiat Oncol. 2025 May 17;34:100786. doi: 10.1016/j.phro.2025.100786 (PMC12150183; doi:10.1016/j.phro.2025.100786)
Supplement: Supplementary Data 2 [file mmc2.docx]

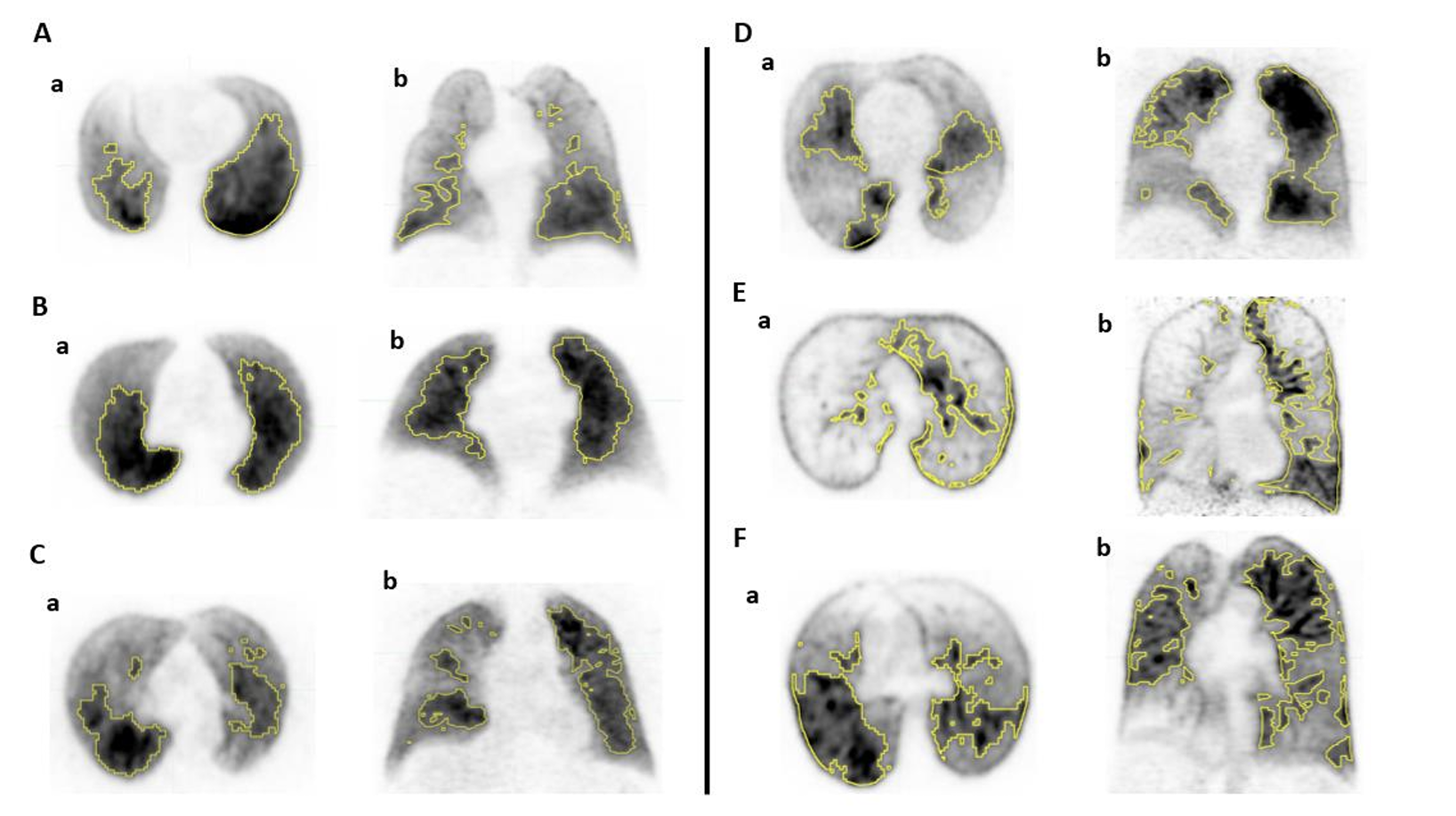


Figure S2. Illustration of perfusion volume FV50% using WLF threshold method in axial (a) and coronal section (b) in patients with normal (A, B and C) and abnormal (D, E and F) pulmonary function tests, adapted from [1].

1. Pinot F, Bourhis D, Bourbonne V, Floch R, Mauguen M, Blanc-Béguin F, et al. New Automated Method for Lung Functional Volumes Delineation with Lung Perfusion PET/CT Imaging. Cancers (Basel). 2023;15:2166.
