# Supplementary material for: Role of functional mapping on Gallium-68 perfusion positron emission tomography and computed tomographic imaging (PET/CT) to assess the risk of long-term radiation-induced lung toxicity after stereotactic body radiation therapy
Source: Phys Imaging Radiat Oncol. 2025 May 17;34:100786. doi: 10.1016/j.phro.2025.100786 (PMC12150183; doi:10.1016/j.phro.2025.100786)
Supplement: Supplementary Data 3 [file mmc3.docx]

A.


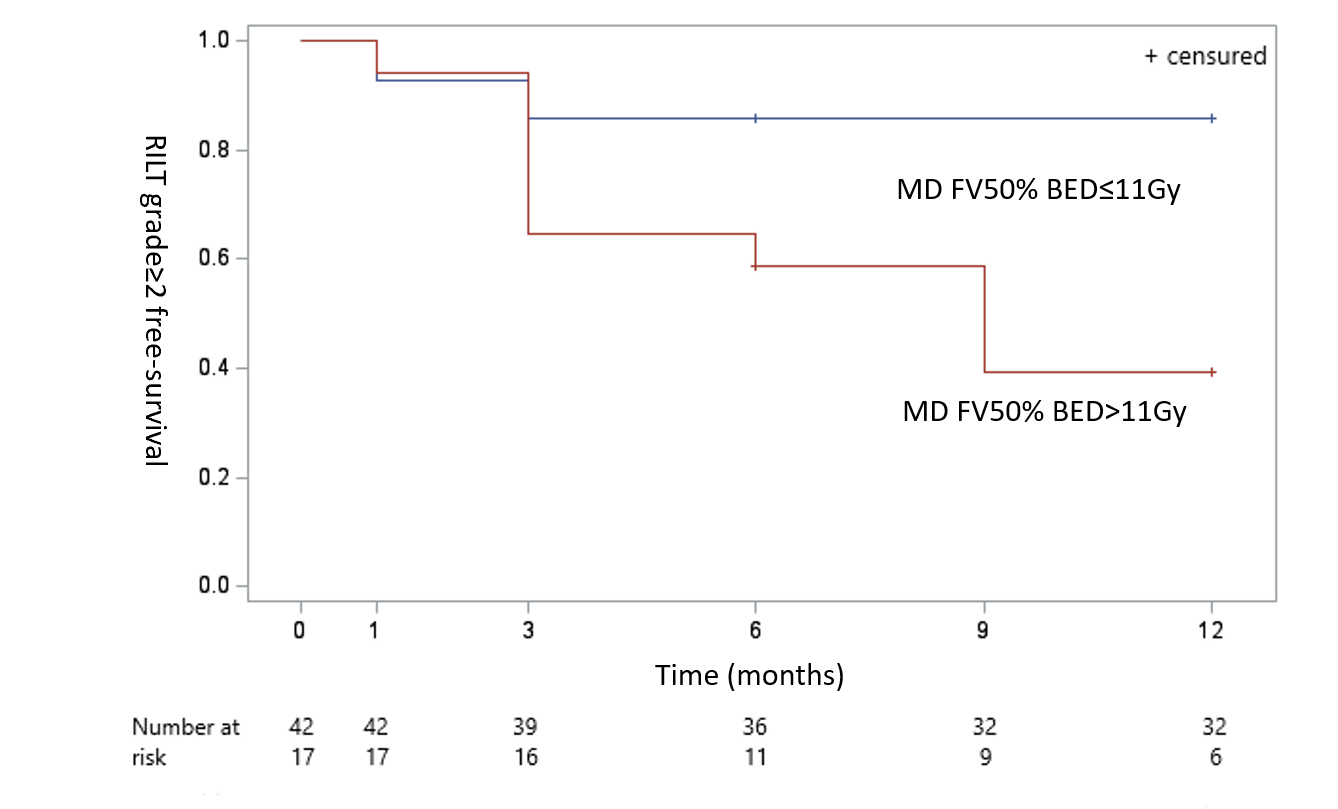


B.


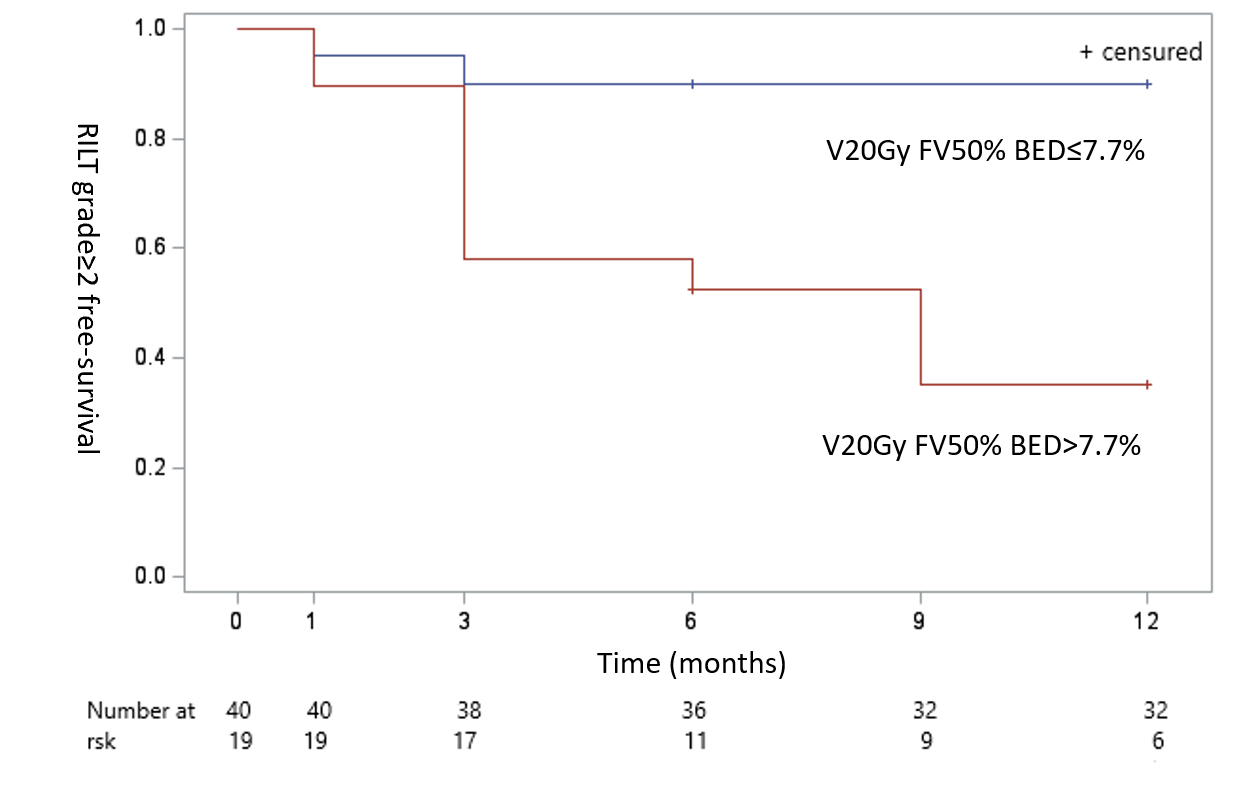


Figure S3 : Risk of ≥grade 2 radiation-induced lung toxicity according to (A) MD FV50% BED (cut-off of 11 Gy) and (B) V20Gy FV50% BED (cut-off of 7.7%) over time.
