# Supplementary material for: Role of functional mapping on Gallium-68 perfusion positron emission tomography and computed tomographic imaging (PET/CT) to assess the risk of long-term radiation-induced lung toxicity after stereotactic body radiation therapy
Source: Phys Imaging Radiat Oncol. 2025 May 17;34:100786. doi: 10.1016/j.phro.2025.100786 (PMC12150183; doi:10.1016/j.phro.2025.100786)
Supplement: Supplementary Data 4 [file mmc4.docx]

**Table S1 : Dosimetric parameters**

|  | **Total (N=59)**  Median (IQR) | **Grade 0-1  (N=49)**  Median (IQR) | **Grade≥2 (N=10)**  Median (IQR) | ***P* value** | **Adjusted p value** |
| --- | --- | --- | --- | --- | --- |
| V10Gy AV (%) | 7.5 (5.3;11.8) | 6.8 (4.5;10.8) | 10.2 (7.5;14.5) | 0.068 | 0.14 |
| V10Gy LFV (%) | 5.2 (1.3;13.7) | 5.3 (1.7;13.8) | 3.0 (1.3;10.6) | 0.56 | 0.65 |
| V10Gy FV50% (%) | 8.1 (3.2;12.4) | 7.2 (2.8;10.4) | 14.3 (13.6;17.6) | 0.0014 | 0.008 |
| V10Gy FV70% (%) | 8.3 (4.5;11.1) | 7.2 (4.4;10.5) | 14.2 (11.0;19.4) | 0.0028 | 0.010 |
| V10Gy FV90% (%) | 8.3 (5.0;11.8) | 7.4 (4.4;10.5) | 13.8 (9.5;15.6) | 0.0097 | 0.024 |
| V10Gy AV (%) BED | 11.1 (8.2;15.2) | 10.5 (8.2;13.7) | 12.4 (10.5;20.1) | 0.15 | 0.25 |
| V10Gy LFV (%) BED | 8.3 (1.9;15.2) | 8.7 (1.9;16.3) | 6.4 (4.2;10.5) | 0.55 | 0.65 |
| V10Gy FV50% (%) BED | 8.8 (5.4;15.3) | 7.6 (4.6;12.7) | 18.1 (15.3;19.5) | <0.001 | 0.005 |
| V10Gy FV70% (%) BED | 10.0 (6.4;15.4) | 9.1 (6.1;12.3) | 17.0 (16.3;19.1) | <0.001 | 0.005 |
| V10Gy FV90% (%) BED | 10.1 (7.4;13.9) | 9.6 (6.9;12.9) | 14.4 (13.4;22.2) | 0.0024 | 0.010 |
| V15Gy AV (%) | 4.6 (3.2;8.1) | 4.2 (2.8;6.7) | 8.4 (4.5;9.4) | 0.11 | 0.20 |
| V15Gy LFV (%) | 3.4 (0.7;9.0) | 3.5 (0.7;9.1) | 0.8 (0.7;6.5) | 0.45 | 0.57 |
| V15Gy FV50% (%) | 5.3 (1.7;8.1) | 3.5 (1.6;6.8) | 9.2 (8.1;13.1) | <0.001 | 0.005 |
| V15Gy FV70% (%) | 5.5 (2.6;7.7) | 4.5 (2.2;6.4) | 9.8 (7.5;13.1) | 0.0026 | 0.010 |
| V15Gy FV90% (%) | 5.6 (2.5;7.5) | 4.7 (2.3;6.6) | 9.8 (5.8;10.5) | 0.014 | 0.031 |
| V15Gy AV (%) BED | 8.5 (5.8;12.8) | 8.3 (5.7;10.5) | 10.8 (8.5;17.0) | 0.10 | 0.20 |
| V15Gy LFV (%) BED | 6.8 (1.6;12.8) | 7.1 (1.3;13.2) | 4.2 (1.6;8.5) | 0.61 | 0.69 |
| V15Gy FV50% (%) BED | 7.2 (3.6;12.1) | 5.6 (3.1;9.0) | 14.1 (11.8;18.2) | <0.001 | 0.005 |
| V15Gy FV70% (%) BED | 7.5 (4.5;11.4) | 7.2 (4.1;10.5) | 14.8 (11.4;15.3) | <0.001 | 0.005 |
| V15Gy FV90% (%) BED | 8.3 (5.4;11.1) | 7.7 (4.5;9.4) | 12.0 (10.9;16.6) | 0.0022 | 0.009 |
| V25Gy AV (%) | 2.0 (1.3;3.8) | 1.9 (1.2;3.3) | 3.0 (1.5;4.9) | 0.22 | 0.34 |
| V25Gy LFV (%) | 1.8 (0.2;4.0) | 1.9 (0.2;4.1) | 0.6 (0.2;2.3) | 0.32 | 0.45 |
| V25Gy FV50% (%) | 2.2 (0.7;3.3) | 1.6 (0.5;3.1) | 4.6 (2.9;6.3) | 0.0018 | 0.009 |
| V25Gy FV70% (%) | 2.1 (1.0;3.9) | 1.9 (1.0;3.0) | 4.9 (2.3;6.3) | 0.0063 | 0.017 |
| V25Gy FV90% (%) | 2.3 (1.2;3.9) | 2.2 (1.0;3.3) | 4.3 (1.8;6.0) | 0.035 | 0.073 |
| V25Gy AV (%) BED | 5.8 (3.9;8.8) | 5.6 (3.6;8.2) | 8.4 (6.0;12.3) | 0.13 | 0.23 |
| V25Gy LFV (%) BED | 4.9 (1.0;10.1) | 5.8 (1.0;10.2) | 2.6 (1.3;6.0) | 0.32 | 0.45 |
| V25Gy FV50% (%) BED | 4.2 (2.0;8.3) | 3.5 (1.6;6.0) | 11.3 (8.3;14.6) | <0.001 | 0.005 |
| V25Gy FV70% (%) BED | 5.0 (2.9;8.1) | 4.4 (2.7;6.9) | 10.1 (8.4;11.4) | <0.001 | 0.005 |
| V25Gy FV90% (%) BED | 5.6 (3.1;8.2) | 4.7 (3.0;6.9) | 9.5 (7.8;12.1) | 0.0043 | 0.013 |
| V30Gy AV (%) | 1.5 (0.9;2.6) | 1.4 (0.9;2.5) | 2.3 (1.1;3.8) | 0.25 | 0.36 |
| V30Gy LFV (%) | 1.2 (0.2;3.0) | 1.4 (0.2;3.1) | 0.5 (0.2;1.4) | 0.37 | 0.50 |
| V30Gy FV50% (%) | 1.5 (0.5;2.4) | 0.9 (0.3;2.4) | 3.6 (1.9;4.9) | 0.0039 | 0.013 |
| V30Gy FV70% (%) | 1.6 (0.7;3.0) | 1.4 (0.6;2.3) | 3.0 (1.8;5.0) | 0.0011 | 0.026 |
| V30Gy FV90% (%) | 1.6 (0.9;2.6) | 1.6 (0.8;2.4) | 2.7 (1.3;4.8) | 0.045 | 0.092 |
| V30Gy AV (%) BED | 5.0 (3.4;8.0) | 4.9 (3.0;7.4) | 7.6 (4.9;10.7) | 0.16 | 0.26 |
| V30Gy LFV (%) BED | 4.3 (0.7;8.7) | 5.3 (0.7;9.3) | 2.0 (1.1;5.0) | 0.32 | 0.45 |
| V30Gy FV50% (%) BED | 3.6 (1.4;7.3) | 2.6 (1.2;5.2) | 10.3 (7.3;12.3) | <0.001 | 0.005 |
| V30Gy FV70% (%) BED | 4.2 (2.2;7.1) | 3.5 (1.8;5.4) | 8.9 (7.8;10.3) | <0.001 | 0.005 |
| V30Gy FV90% (%) BED | 4.8 (2.4;6.7) | 3.8 (2.3;6.2) | 8.8 (6.2;10.9) | 0.0051 | 0.015 |

Abbreviations IQR=Interquartile range; AV= Anatomical volume ; MLD=Mean dose to lung volume ; LFV= Low functional volume ; FV=Functional volume ; VxGy=percentage of lung volumes receiving x Gy ; BED=Biologically effective dose
